# Cardiovascular Diseases Among Kidney Transplant Recipients at National Guard Hospital—Jeddah

**DOI:** 10.3390/healthcare14080987

**Published:** 2026-04-09

**Authors:** Nadia O. Elamin, Hala E. Danish, Razan O. Bawazir, Renad F. Alharthy, Renad I. Katib, Joud M. Alharthy, Maryam N. Alotibi, Turki A. Banamah

**Affiliations:** 1 College of Medicine, King Saud bin Abdulaziz University for Health Sciences, Jeddah 21423, Saudi Arabia; 2 King Abdullah International Medical Research Center, Jeddah 22384, Saudi Arabia; 3 Ministry of the National Guard-Health Affairs, Jeddah 11426, Saudi Arabia; 4 Nephrology Section, Department of Medicine, Ministry of the National Guard-Health Affairs, Jeddah 22384, Saudi Arabia

**Keywords:** cardiovascular disease, cardiovascular risk factors, kidney transplant recipients, medication compliance, post-transplantation

## Abstract

**Background:** Cardiovascular disease (CVD) represents the second leading cause of death among kidney transplant recipients (KTRs). CVD risks post-transplantation increase with aging, obesity, dyslipidemia, diabetes, hypertension, inactivity, sleep disturbances, immunosuppressant medications use, and graft dysfunction. This study assessed CVD prevalence and risk factors among KTRs. **Methods:** A cross-sectional study was conducted at National Guard Hospital, Jeddah between 2012–2022. Information was collected from the patients’ medical records. Physical activity, sleep, and adherence to immunosuppressant therapy were evaluated via interviews with adult KTRs using the International Physical Activity Scale, Jenkins Sleep Scale, and Immunosuppressant Therapy Barrier Adherence Scale, respectively. **Results:** Sixty-four KTRs were included: 67% were males, and the median age was 44.7 years. Eighteen patients (28.1%) had CVD, and 61.1% of them developed ischemic heart disease. KTRs with CVD were older, had lower estimated glomerular filtration rate (eGFR), and higher Hemoglobin A1c (HbA1c), but these differences were not statistically significant (*p* > 0.05). Patients with CVD had significantly lower LDL (*p* = 0.02) and more aspirin and statin use (*p* < 0.05). Forty-five patients (70.3%) completed the interview; most of them had few sleep disturbances and good adherence to immunosuppressant therapy. Low physical activity was reported by KTRs with CVD. **Conclusions:** CVD was present in over one-quarter of KTRs. Patients with CVD were older, less active, had lower GFR, higher HbA1c, and significantly lower LDL. More use of aspirin and statin improved the glycemic control, physical activity, and medication adherence, and may help in reducing CVD burden among KTRs.

## 1. Introduction

The physiological functions of the kidneys include elimination of waste products while maintaining blood pressure, electrolyte, and fluid balance. When the functional capacity of the kidneys diminishes, accumulation of the waste products together with disturbance in blood pressure, electrolyte, and fluid balance occur, leading to renal failure [1]. Renal transplantation is the most prevalent form of organ transplant performed to treat chronic end-stage kidney disease. Renal transplantation greatly enhances renal outcomes and hinders the development of cardiovascular disease (CVD), which is a leading cause of mortality in patients with renal diseases [2,3]. Despite the significant improvements in survival and quality of life after transplantation, CVD continues to be the second most common cause of death among kidney transplant recipients (KTRs) [2,3].

CVD includes hypertensive heart diseases, ischemic heart diseases (IHDs), heart failure, peripheral arterial disease, and other cardiac and vascular conditions [4]. Post-transplant CVD can be caused by preexisting or acquired traditional CVD risk factors like obesity, dyslipidemia, hypertension (HTN), left ventricular hypertrophy, and diabetes mellitus (DM) [5]. Also, some transplant-specific factors were linked with increased cardiovascular risk, such as proteinuria, recurrent infections, and poor graft function [5]. Patients need to comply with their immunosuppressant medication post-transplant to avoid transplant dysfunction. The use of immunosuppressant medications and other lifestyle factors had major roles in affecting the other existing risk factors [6]. After transplantation, the use of immunosuppressant therapy (IT) has been shown to deteriorate preexisting HTN and DM [1,5].

This study aimed to estimate the prevalence of CVD among recipients after kidney transplantation and to assess some important CVD risk factors. Moreover, the adherence to immunosuppressant therapy, patients’ physical activity, and their sleep were also evaluated.

## 2. Method

A cross-sectional, single-center study was conducted at the National Guard Hospital in King Abdullah Medical City in Jeddah (KAMC-JD). The study was carried out in accordance with the Code of Ethics of the World Medical Association (Declaration of Helsinki). All adult kidney transplant recipients (KTRs) who underwent transplantation between 2012 and 2022 were included and assessed for cardiovascular disease (CVD). Physical activity, sleep, and adherence to immunosuppressants were subsequently evaluated in patients who agreed to participate in the interview. All adult renal transplant recipients were invited through phone calls to participate in the survey and were informed about the study aims to obtain their verbal consent before completing the interview. Participation in the surveys was voluntary, and the patients had the right to withdraw at any time. Patients under 13 years and those who underwent their transplants before 2012 were excluded. The electronic medical records (Best-Care system 2.0) were reviewed to obtain patients’ demographic and medical data, including age, sex, past medical history, the type of transplant and duration since transplant, the last lipid profile and hemoglobin A1c (HbA1c) levels, the medications used (anti-platelets, lipid lowering, and immunosuppressant agents), Body Mass Index (BMI), and information about any CVD after transplantation (according to the International Classification of Diseases 11th Revision). The standard immunosuppressive protocol used for induction therapy included thymoglobulin for patients at high risk of rejection and basiliximab for those at low risk. For maintenance therapy, most patients received a combination of at least three immunosuppressive agents, typically including cyclosporine and prednisone, along with either tacrolimus or mycophenolate mofetil (e.g., CellCept). The dosage and number of medications were individualized based on therapeutic drug monitoring, particularly blood drug levels, with most agents administered twice daily.

Patients who agreed to participate in the study were asked to complete a survey based on three scales: the International Physical Activity Scale (IPAS), the Jenkins Sleep Scale (JSS), and the Immunosuppressant Therapy Barrier of Adherence Scale (ITBS) [7,8,9]. All the questions in the survey were translated into Arabic by an Arabic-speaking linguist to guarantee participants’ comprehension. The IPAS was used to assess the type and intensity of physical activity within one week and to find the estimated total physical activity in metabolic equivalent tasks (METs in minutes per week) [7]. The ITBS is a validated instrument commonly used to identify the barriers and causes of immunosuppressant therapy (IT) noncompliance among KTRs [8]. The barriers to IT compliance assessed include: taking too many tablets per day; taking too many medications at the same time; missing the dose if they had side effects; not having enough money; having a change in routine; being depressed, confused, not feeling good; not remembering it. The responses ranged from agree, to neutral, to disagree [8]. Regarding sleep, using JSS, four categories of sleep were assessed during the last month: waking up several times per night, trouble falling asleep, staying asleep, and waking up tired and worn out. The responses of patients for each category during the last month were added. Two categories with high and low frequency of sleep disturbances during the last month were defined, with scores above 11 representing a high frequency of sleep disturbances (HSDs) [9]. Confidentiality was secured, and all data was saved and only accessible to the research team.

Statistical analysis was performed using John’s Macintosh Project (JMP®), version 10 (SAS Institute Inc., Cary, NC, USA). The Shapiro–Wilk test was used to determine the normality of the continuous variables. The normally distributed variables (parametric) were presented as means and standard deviation [mean ± SD], and non-normally distributed variables (non-parametric) were presented using [median (IQR)]. The parametric variables were compared using the independent samples *t*-test, while non-parametric ones were compared using the Wilcoxon rank-sum test. Categorical variables were expressed as percentages and frequencies [n (%)], and the Chi-square test was used to compare the groups. *p*-values less than 0.05 were considered statistically significant.

## 3. Results

Eighty-four kidney transplant recipients (KTRs) were identified. All relevant data were extracted from the patients’ medical records. Sixty-four KTRs were included in the current study, while 20 patients were excluded due to missing data (23.8%). Participants were predominantly male (67%). Most transplants occurred at KAMC-JD, and the majority of patients received kidney transplants from living relative donors (60.9%). All patients received at least three medications as part of maintenance therapy, including cyclosporine and prednisone, in combination with either tacrolimus, which was prescribed in most patients (98.4%), or mycophenolate mofetil (e.g., CellCept). Among lipid-lowering therapies, atorvastatin was the most frequently used, reported in 39 patients (60.9%).

CVD was present in 18 patients (28.1%): eleven developed ischemic heart diseases (61.1%), four had high blood pressure (22.2%), two had left ventricular hypertrophy (11.1%), and one had a myocardial infarction (5.6%). Participants were divided into two groups according to the presence or absence of CVD to assess the association between CVD and risk factors (Table 1). BMI was equivalent in both groups. Patients with CVD were older and had lower cholesterol and estimated glomerular filtration rate (eGFR) compared to patients without CVD; however, these differences were not statistically significant (*p* > 0.05). Patients with CVD had a higher mean HbA1c compared with the other group, but this difference was not statistically significant (*p* = 0.15). The LDL levels were significantly lower in patients with CVD (*p* = 0.02), and they had statistically significant more statin (*p* < 0.0001) and aspirin use (*p* = 0.04) compared to patients with no CVD (Table 1). The median transplant duration was 5.0 years (IQR: 4) in patients with CVD and 5.0 years (IQR: 5.25) in patients without CVD; no statistically significant difference was observed between the two groups. Less than half of patients with no CVD were using statins. Among patients without CVD, those not using statins had higher LDL levels (median 3.29 mmol/L, IQR 1.30; n = 27) compared to those using statins (median 1.93 mmol/L, IQR 0.78; n = 14), with a statistically significant difference (*p* < 0.0001).

**Figure 1:**
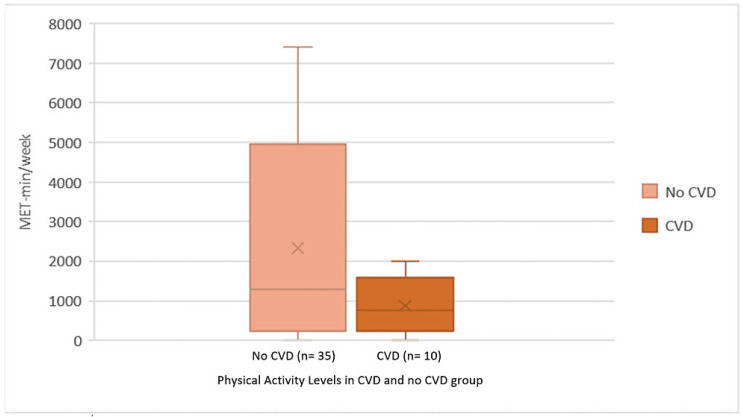
Physical activity after transplant in patients with CVD and those with no CVD. The physical activity was assessed after transplant in patients who developed CVD (n = 10) and those with no CVD (n = 35) by the International Physical Activity Scale using metabolic equivalent tasks in minutes per week (METs min/week).

**Figure 2:**
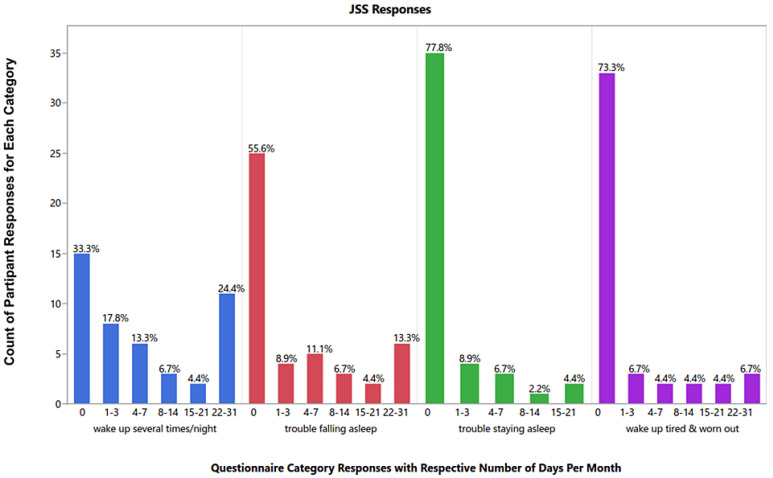
Sleep assessment in patients after transplant using the Jenkins sleep scale (n = 45). Using the Jenkins sleep scale (JSS), four categories of sleep were assessed during the last month: waking up several times per night, trouble falling asleep, staying asleep, and waking up tired and worn out. The count and percentage of patients having problems in the four categories for 0, 1–3, 4–7,8–14, 15–21 or 22–31 times during the last month was reported. Most patients had no or few problems in the four categories. A quarter of KTRs reported waking up several times in the night for 22–31 days, but only three (6.7%) woke up exhausted more than 22 times during the last month. There were no significant differences in sleep disturbances between patients with and without CVD (all *p*-values were >0.05).

**Figure 3:**
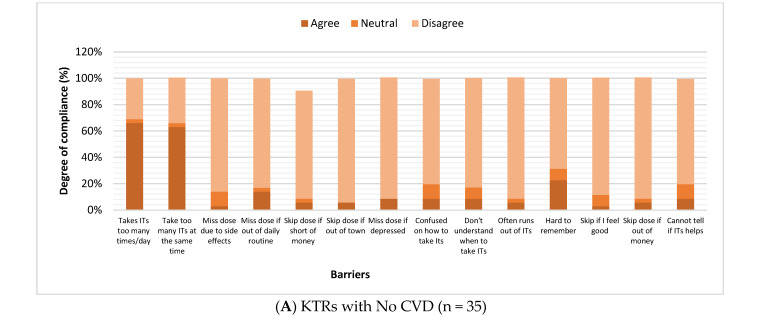

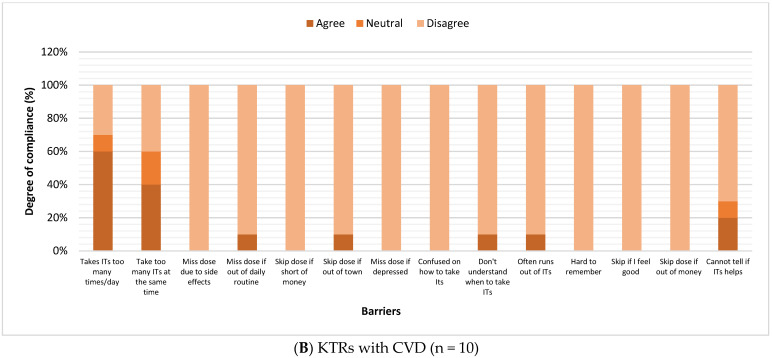
Medication compliance among kidney transplant recipients. The Immunosuppressant Therapy Barrier of Adherence Scale was used to assess compliance to immunosuppressant therapy (IT) among kidney transplant recipients (KTRs) ((**A**) with no CVD (n = 35), (**B**) with CVD (n = 10)). The common barriers to compliance were assessed, with responses ranging from agree, to neutral, to disagree. Taking too many tablets per day, and too many medications at the same time were the only barriers that most patients agreed with but they don’t agree with most of the other barriers. There were no significant differences in medication adherence or reported barriers between KTRs with and without CVD, indicating that the presence of CVD did not substantially affect compliance (all *p*-values were >0.05).

Forty-five patients answered the survey questions regarding physical activity, sleep, and immunosuppressant compliance, with a response rate of 70.3%. Lower levels of physical activities were reported by patients with CVD (Figure 1). Regarding sleep, few patients reported having problems in the four sleep categories: waking up several times per night, waking up tired and worn out, trouble falling asleep, or staying asleep. A quarter of the KTRs (24.4%) reported waking up several times in the night for 22–31 days, but only three (6.7%) woke up exhausted for 22–31 days. None of the KTRs had trouble staying asleep during the night for more than 21 days during the last month. The frequency of sleep disturbance categories among KTRs was assessed using the Jenkins sleep scale scores, comparing CVD with the other group, with no significant difference detected (Figure 2). The frequency of sleep disturbance was further categorized by the sum of JSS scores, where having scores above 11 represents a high frequency of sleep disturbances (HSDs). Most patients experienced low frequency of sleep disturbance (88.9%) (Table 2). Only five participants experienced HSD; two patients from the CVD group compared with three KTR with no CVD, and the difference between the two groups was not significant (*p* = 0.30) (Table 2). Almost 70% of KTRs who responded to the survey were compliant with their medications. The majority of patients never missed their medicines if they were depressed, feeling good, or did not remember it. The most common barrier to immunosuppressant compliance was taking too many tablets per day, where KTRs with no CVD and with CVD agreed at rates of 37% and 40%, respectively (Figure 3). There were no significant differences in medication adherence or reported barriers between KTRs with and without CVD, indicating that the presence of CVD did not substantially affect compliance. Yet, most patients with no CVD (60%) agreed that taking too many medications at the same time is a barrier, compared with 40% of the KTRs in the CVD group. Most patients in both groups (those without CVD and those with CVD) reported continued medication use despite potential barriers, including experiencing side effects (63% vs. 80%), financial constraints (63% vs. 80%), and changes in daily routine (54% vs. 70%), respectively.

## 4. Discussion

In the current study, from 64 kidney transplant recipients (KTRs) included, 18 patients (28.1%) had CVD. IHD was the most prevalent condition, affecting 61.1% of patients with CVD. In a previous study including 770 KTRs, most of them were referred to the cardiology department, and 8.6% of them developed a cardiovascular event within 3 months (defined as a composite of cardiac death, myocardial infarction, coronary revascularization, or heart failure necessitating admission within 3 months of transplantation) [10]. Differences in study design, population characteristics, diagnostic approaches, baseline cardiovascular risk factors, duration after transplantation, and the intensity of cardiovascular screening may have contributed to and may explain the discrepancy observed in the present cohort. Nearly all KTRs had preexisting hypertension, which may worsen following transplantation as a consequence of immunosuppressant therapy [5]. In the current study, including hypertension may overestimate prevalence of CVD, but having high pressure after treatment will increase the risk for IHD and other CVD, and can also affect the graft function and overall patients’ survival. In the current study, elevated blood pressure was observed in approximately one-third of KTRs, with 22% having hypertension and 11.1% demonstrating left ventricular hypertrophy. High blood pressure could further increase the risk of IHD and myocardial infarction among this population. Therefore, blood pressure lowering is necessary in the primary prevention of CVD after transplantation. A reduction of each mm Hg in systolic blood pressure (above 110 mm Hg) can reduce the CVD risk by 2–3% [11]. In addition to pharmacologic management, early identification and control of modifiable risk factors are essential for the effective management of post-transplant hypertension.

In the current study, patients with CVD were older, which is comparable to other studies in which age was a risk factor for overall mortality after kidney transplants [3]. Our findings revealed a slightly higher mean BMI in the KTRs with CVD (*p* = 0.95). However, obesity did play a major role in increasing the cardiac risk and was independently associated with graft failure and long-term graft dysfunction in other studies [12]. Dyslipidemia is a common finding after transplant that increases the risk for CVD, especially IHD. In the present study, patients with CVD had lower total cholesterol and significantly lower mean LDL compared to patients who did not develop CVD. This can be explained by the more frequent use of medications like statins and aspirin among patients with CVD. In the existing study, a higher mean HbA1c approaching diabetic levels was found in KTRs with CVD compared to patients without CVD. It was reported that transplanted patients with diabetes had markedly increased cardiac risk, approaching 2 to 5 times the risk in non-diabetic patients [12]. Glycemic control with lifestyle interventions and pharmacotherapy before and after transplantation are necessary to reduce CVD risk. Some transplant-specific factors, like proteinuria and poor graft function, were linked to a higher risk of CVD [5]. Lower eGFR was found in KTRs who developed CVD compared to the other group. Renal graft dysfunction is a prominent independent risk factor for CVD that can worsen all other risk factors. Preserving the renal function can lower the incidence of cardiac events and improve graft survival and overall survival. Many studies have shown a significant association between proteinuria and lower survival rates among KTRs, where patients with no proteinuria had higher survival rates [13]. Even a low level of proteinuria was correlated to a worse prognosis [12]. In the current study, no difference in the level of proteinuria was found between patients with and without CVD, most likely due to the common use of rennin-angiotensin-aldosterone system blockers, which can reduce proteinuria levels [12]. The median duration time after transplant was 5 years, with no significant difference between the two groups. A potential limitation of this study is the risk of a Type II error (β), whereby the associations between variables may not have been detected because of the limited sample size and inherent variability in physiological measurements, which may have reduced the statistical power of the analysis and hindered the detection of modest but clinically relevant effects.

Reduction of cardiac risk after transplant could be achieved by lifestyle modification, especially smoking cessation and exercise [12]. A previous study demonstrated that smoking increases cardiac risk and mortality rates by up to 70% in KTRs [14] However, in the present study, more smokers were found among patients with no CVD; we hypothesize that KTRs who had already developed CVD probably had better awareness about smoking as an independent risk factor for CVD, graft rejection, and mortality.

The estimated percentage of myocardial infarction attributable to physical inactivity in the INTERHEART study was 12% [15]. In the current study, KTRs with CVD reported having low levels of physical activity. Generally, KTRs had reduced peak aerobic capacity, muscle strength, arterial function, and an unfavorable CVD risk profile [15,16]. It was recommended that supervised exercise could be incorporated into the treatment plans before and after a kidney transplant [17]. Interventions like aerobic or resistance exercises, or a combination of both, were used for KTRs with no observed harm [17]. The risk for CVD generally increases with sleep disorders. Some studies found more disturbed functional sleep among KTRs than among control subjects [18,19]. In the present study, sleep quality was not greatly disturbed in most KTRs. A potential response bias might result from having 30% of KTRs not responding. The non-respondents may have more problems with sleep, physical activity, and medication compliance. The most-reported sleep issue was waking up several times per night, while in another study the predominant sleep problems were difficulty staying asleep and problems falling asleep [20]. Our findings may differ because of the different scales used (JSS instead of the Pittsburgh Sleep Quality Index (PSQI)). Moreover, a large discrepancy in sleep was found after renal transplant. Some studies reported better sleep after transplant, depending on the graft function [21]. The pathophysiology of sleep disorders post-transplant includes anxiety of losing the graft, immunosuppressant medications use, and the presence of other comorbidities like obesity, CVD, or malignancies [21]. On the other hand, it was hypothesized that improving renal function after transplant can elevate melatonin levels, resulting in amended sleep [22].

Medication noncompliance was linked with education level, financial status, literacy level, and patients’ beliefs [23]. In the current study, the most common obstacle to compliance was taking too many doses per day. Another study reported that most patients agreed that taking too many doses per day and taking many tablets at once were the two most common “uncontrollable barriers” [24]. Consistent with a previous study, our findings showed that patients did not skip doses when depressed, out of town, confused about their medication, or thought their medication did not help them [24]. This reflects that the patients were aware of the importance of adherence. Moreover, in the current study, KTRs were granted access to most of their medication, which explains why the majority did not miss doses due to financial issues. On the other hand, other studies disclosed that many patients had several barriers to medication adherence [25]. Although there were no significant differences in medication adherence or reported barriers between KTRs with and without CVD, slightly higher adherence was noticed among patients with CVD, which may be explained by an increased perceived disease severity. Long-term non-adherence to medication was associated with graft loss, depression, anxiety, and stress [20]. Continuous counselling before and after transplantation on noncompliance risks should be implemented, with regular follow-up to ensure that the patients follow instructions [26]. Fortunately, multiple counselling sessions about the importance of compliance are usually provided by our institute (before and after transplantation). The recommended use of a simplified once-daily medication regimen can reduce total percentages in some ITBS sub-scales, including “uncontrollable factors and can improve compliance and long-term graft function in KTRs” [26].

## 5. Limitations

Among the limitations of this study is the cross-sectional, single-center design, which limits the ability to establish causal relationships and may restrict the generalizability of the findings. Second, there is a potential risk of a Type II error, whereby true associations may not have been detected due to the relatively small sample size, partly resulting from the exclusion of patients with incomplete or missing data. This may have reduced the statistical power to detect significant differences between groups and hindered the identification of modest but clinically relevant effects. Therefore, non-significant findings should be interpreted with caution. In addition, the inclusion of hypertension as part of the cardiovascular disease (CVD) definition may have led to an overestimation of CVD prevalence. Furthermore, challenges in reaching patients by telephone for the survey component resulted in a response rate of 70%, and the reliance on self-reported data may have also introduced some reporting bias.

Despite these limitations, one of the study’s strengths is that, to our knowledge, it is the first study in Saudi Arabia to explore the risk factors of CVD in post-renal transplant patients in relation to physical activity, sleep, and medication adherence. Future multicenter studies with larger sample sizes can enhance the statistical power and improve the generalizability of the findings. Moreover, assessing more CVD risk factors, like anemia, low vitamin D, high uric acidic, and vascular calcification before and after transplant, can help in understanding cardiovascular risk.

## 6. Conclusions

In conclusion, approximately a quarter of KTRs in this study had CVD. Patients who developed CVD tended to be older, had poorer glycemic control, lower eGFR, and lower levels of physical activity, although most of these differences did not reach statistical significance, probably due to the relatively small sample size. The significantly lower LDL levels observed in patients with CVD may be attributed to their more frequent use of statins and aspirin, indicating increased levels of perceived disease severity rather than reflecting a true protective pattern. Good adherence to immunosuppressant therapy and few sleep disturbances were observed, with no significant differences between groups.

Although causal relationships cannot be established given the cross-sectional design of the study, reducing the incidence of CVD post-transplantation requires a comprehensive individualized treatment plan emphasizing healthy lifestyle, physical activity, optimal glycemic control, and adherence to lipid-lowering, anti-hypertensive, and immunosuppressant medications. Future multicenter studies with larger sample sizes are needed to better establish causal relationships and optimize long-term outcomes in this high-risk population.

## Figures and Tables

**Table 1:** Comparison of some risk factors between kidney transplant recipients who had CVD and those with no CVD.

Variables	Group 1 (No CVD) n = 46 (71.9%)	Group 2 (CVD) n = 18 (28.1%)	*p*-Value
Age (years)	40 (44.75) [CI: 29.6–50.4]	55 (18.25) [CI: 48.2–61.8]	0.06
Sex			
Male	31 (48.4%)	12 (18.8%)	0.96
Female	15 (23.4%)	6 (9.3%)	
Donor type			
Deceased donor	5 (7.8%)	0 (0%)	0.30
Living unrelated donor	13 (20.3%)	7 (10.9%)	
Living relative donor	28 (43.6%)	11 (17.2%)	
Duration since kidney transplant (years)	5.0 (5.25) [CI: 2.0–9.0]	5.0 (4) [CI: 4.0–7.0]	0.922
Smoking	6 (9.4%)	1 (1.6%)	0.39
Aspirin	13 (20.3%)	10 (15.6%)	0.04
Statins	19 (29.7%)	16 (25%)	<0.0001
Body mass index (Kg/m^2^)	28.36 ± 1	28.47 ± 1.6	0.95
Hemoglobin A1c (g/dL)	5.85 (1.3) [CI: 5.55–6.15]	6.3 (1.9) [CI: 5.60–7.00]	0.15
Total cholesterol (mmol/L)	4.98 ± 0.15	4.47 ± 0.25	0.09
LDL (mmol/L)	2.96 (1.56) [CI: 2.60–3.32]	2.1 (1.36) [CI: 1.60–2.60]	0.02
HDL (mmol/L)	1.21 ± 0.33	1.24 ± 0.35	0. 74
Triglycerides (mmol/L)	1.19 (0.57) [CI: 1.06–1.32]	1.52 (1.17) [CI: 1.09–1.95]	0.13
Proteinuria level (mg/dL)	68 (20.25) [CI: 63.3–72.7]	68 (18.75) [CI: 61.1–74.9]	0.70
eGFR (mL/min)	76.76 ± 3.93	65.30 ± 6.29	0.13

Parametric continuous variables were reported as [mean ± SD], non-parametric continuous variables as [median (IQR)] CI: 95% confidence intervals, and qualitative data as [n (%)]. The independent *t*-test, Wilcoxon (Mann–Whitney U) test, and chi-square test were used, respectively, to compare the groups. A *p*-value < 0.05 was considered statistically significant. CVD: cardiovascular disease, eGFR: estimated glomerular filtration rate, HDL: high-density lipoprotein, LDL: low-density lipoprotein.

**Table 2:** Frequency of sleep disturbances among KTRs with CVD and those with no CVD.

	CVD (n = 10)	No CVD (n = 35)	Total	*p*-Value
High frequency of sleep disturbances	2 (20%)	3 (8.57%)	5 (11.1%)	0.306
Low frequency of sleep disturbances	8 (80%)	32 (91.43%)	40 (88.9%)	

The frequency of sleep disturbance among KTRs was categorized by the sum of the Jenkins sleep scale scores. During the last month four categories of sleep were assessed: waking up several times per night, trouble falling asleep, staying asleep, and waking up tired. After calculating the total score for each patient, those with scores above 11 were grouped as having high frequency of sleep disturbances. Chi-square test was used to compare between the groups. (A *p*-value < 0.05 was considered significant).

## Data Availability

The data presented in this study are available on request from the corresponding author due to privacy and ethical restrictions.

## Abbreviations

BMI: Body Mass Index, CVD: Cardiovascular disease, DM: diabetes mellitus, eGFR: estimated glomerular filtration rate, HbA1c: hemoglobin A1c, HDL: high-density lipoprotein, HTN: hypertension, IHD: ischemic heart disease, IPAS: International Physical Activity Scale, IT: immunosuppressant therapy, ITBS: Immunosuppressant Therapy Barrier of Adherence Scale, JSS: Jenkins sleep scale, KAIMRC: King Abdullah International Medical Research Center, KAMC-JD: King Abdullah Medical City in Jeddah, KTRs: kidney transplant recipients; LDL: low-density lipoprotein, METs: metabolic equivalent tasks, PSQI: Pittsburgh Sleep Quality Index.
